# Supplementary material for: Discovery of Alpha-Gal-Containing Antigens in North American Tick Species Believed to Induce Red Meat Allergy
Source: Front Immunol. 2019 May 17;10:1056. doi: 10.3389/fimmu.2019.01056 (PMC6533943; doi:10.3389/fimmu.2019.01056)
Supplement: Supplementary Figure 1 — Screening of proteins from Amblyomma maculatum for α-gal. Unfed and partially-fed gut tissue and salivary gland tissue homogenates and saliva were run using (A) 12.5% SDS-PAGE, and (B) western blot using anti-gal IgM antibody. Lane 1: A broad range (11–245 kDa) pre-stained protein standard, Lane 2: Am. maculatum unfed midgut tissue, Lane 3: Am. maculatum 3D partially-fed midgut tissue, Lane 4: Am. maculatum partially-fed 8D midgut tissue, Lane 5: Am. maculatum unfed salivary glands, Lane 6: Am. maculatum 3D partially-fed salivary glands, Lane 7: Am. maculatum 8D partially-fed salivary glands, Lane 8: Am. maculatum 8D saliva, Lane 9: Bovine serum albumin and, Lane 10: Diluted sheep blood. [file Data_Sheet_1.docx]

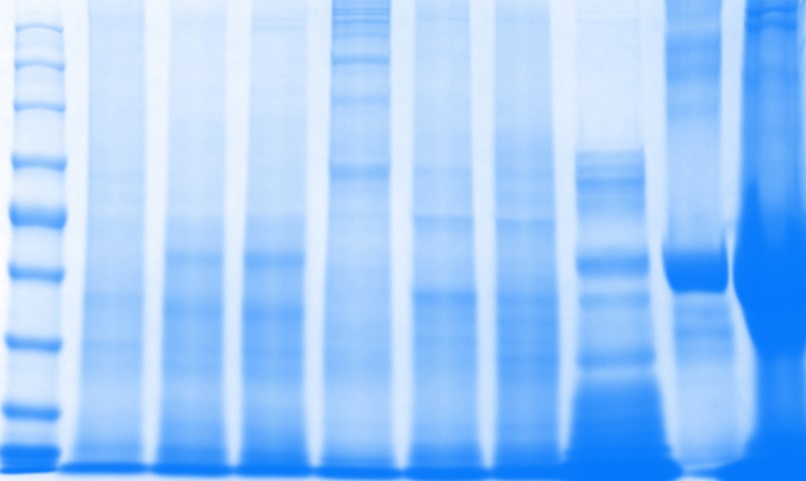

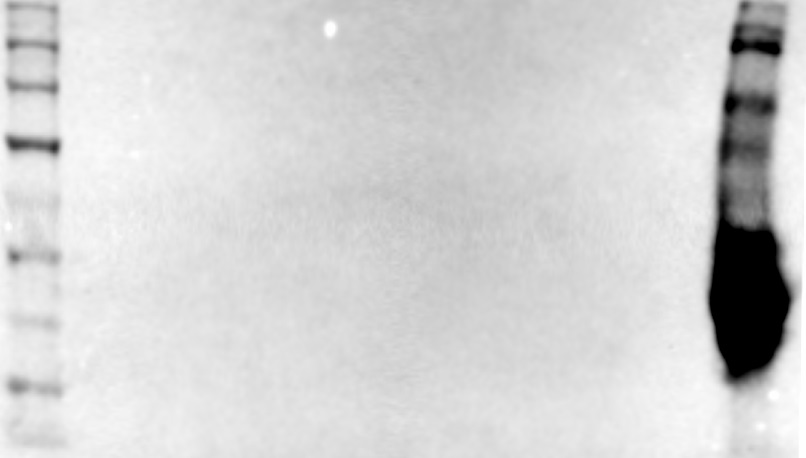


**1 2 3 4 5 6 7 8 9 10**

**A**

**B**

**MW (kDa)**

**100 -**

**80 -**

**245 -**

**190 -**

**135 -**

**58 -**

**46 -**

**32 -**

**25 -**

**100 -**

**80 -**

**245 -**

**190 -**

**135 -**

**58 -**

**46 -**

**32 -**

**25 -**

**Supplementary Figure 1 – Screening of proteins from *Amblyomma maculatum* for α-gal.** Unfed and partially-fed gut tissue and salivary gland tissue homogenates and saliva were run using **A)** 12.5% SDS-PAGE, and **B)** western blot using anti-gal IgM antibody. Lane 1: A broad range (11–245 kDa) pre-stained protein standard, Lane 2: *Am. maculatum* unfed midgut tissue, Lane 3: *Am. maculatum* 3D partially-fed midgut tissue, Lane 4: *Am. maculatum* partially-fed 8D midgut tissue, Lane 5: *Am. maculatum* unfed salivary glands, Lane 6: *Am. maculatum* 3D partially-fed salivary glands, Lane 7: *Am. maculatum* 8D partially-fed salivary glands, Lane 8: *Am. maculatum* 8D saliva, Lane 9: Bovine serum albumin and, Lane 10: Diluted sheep blood.


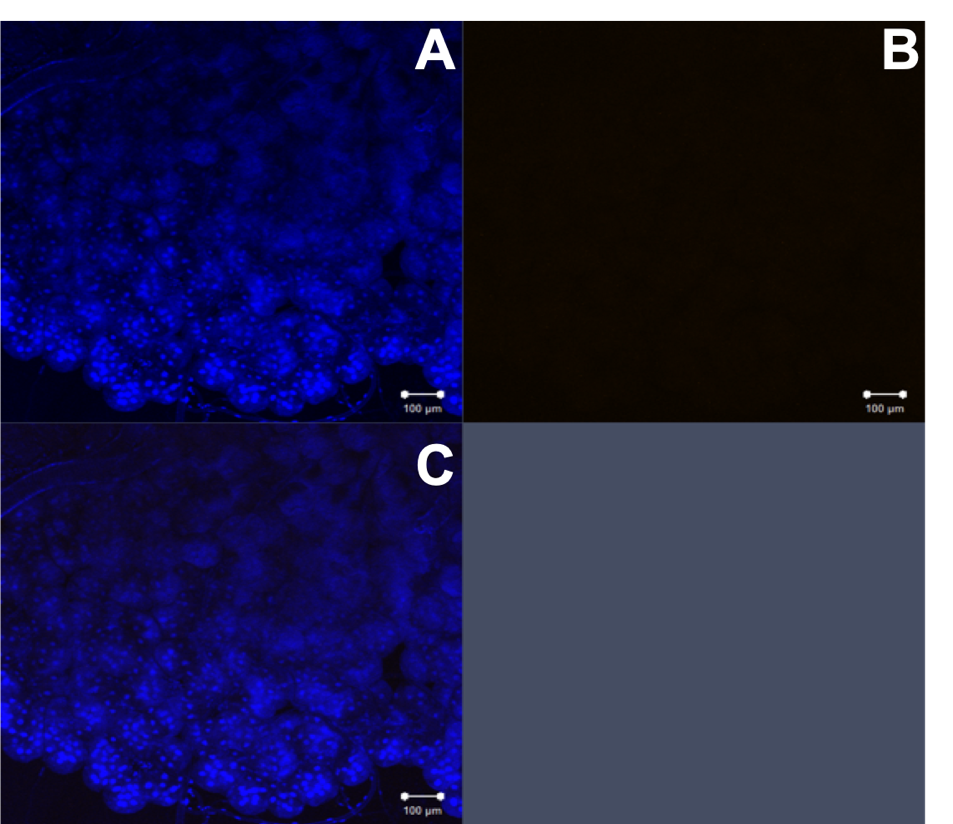


**Supplementary Figure 2 – α-gal immunolocalization in partially-fed *De. Variabilis salivary glands.*** *De. variabilis* salivary gland images (20×) using DAPI (A), alpha-gal IgM (B), and merged images (C).
